# The Futility of Flogging

**Published:** 1921-03-19

**Authors:** 


### THE PUBLIC WELL-BEING.

### The Futility of Flogging.

### AN "UNSENTIMENTAL" ANALYSIS.

An active discussion is going on in the newspapers on the subject of caning in schools. Periodically the question is raised, and on two sides are ranged those who take a merely sentimental view and are prepared to oppose anything which raises in their minds the obnoxious idea of pain, and those who, because they attended in their youth schools which throve on flogging, think that there is some virtue in corporal punishment which makes hardy men. The question is an old one. Traditionally the schoolmaster's sign of office was the birch, and in the Middle Ages whipping at the cart was one of the commonest forms of punishment.

But the protests against such methods of punishment are almost as old as they themselves are, and there are two sides to the question which ought to be discussed with far more of the scientific spirit than usually plays about them. The members of the medical profession are engaged most of their lives in inflicting pain in various degrees, and to say to them merely that corporal punishment is painful, and therefore bad, does not carry conviction. They inflict pain to avoid greater pain, and so on this point their minds are open. It is significant, again, that the discussion on caning in schools is accompanied by or has been followed by the discussion of flogging as a cure for or a deterrent of crime. The association is not a casual one. The advocacy of caning and flogging arises from a point of view, and it is the soundness of this attitude, and not the rights and wrongs of particular cases, which is the real matter of concern. A tap with a cane may be an affair equally meaningless to master and pupil, but those who really believe in the efficacy of this method meant by it something far more serious.

Public-school flogging is often praised because it makes boys "hard," but if it has this effect it is proving ineffective for its true purpose, because it is making boys indifferent and less likely to fear that kind of punishment. Even flogging as carried out under the law has proved no real deterrent, for there are numerous cases of men who have been soundly flogged being brought up again for exactly the same offence, and being sentenced to prison because in the opinion of their judges flogging had failed to deter them from their crimes. It is also a matter for serious reflection that in flogging schools the practice never seemed to do away with the necessity for itself, but rather to grow in intensity; and it is, of course, well known that boys who at their first sight of flogging broke down in tears or nearly fainted have come in later years actually to receive a flogging themselves with something almost like equanimity. There are magistrates who believe that flogging puts down a certain kind of crime, but there are many others, especially those who have followed up the results of their sentences, who believe that it is practically useless. In any case, flogging has its limiits. Formerly women were flogged, and in recent years cases can be quoted of magistrates almost prepared to revert to such a practice; but the general attitude is completely opposed to the flogging of girls or women, and it does not seem a great step to carry the argument forward in favour of its abolition for men and boys. Corporal punishment would seem to be opposed to all the modern views of psychological methods. It merely overlays wrongdoing, instead of probing it and analysing it with a view to the removal of the cause. It is at best a short cut, a method of avoiding trouble or of seeking to avoid it. Perhaps its real value, if it can be said to have a real value, is the relief it gives to the feelings of the administrator, expressed in one of Mr. Bernard Shaw's half-puzzling remarks when he said that one should certainly never strike a child except in anger.

This remark, indeed, penetrates to one of the roots of the matter. Psychologists have long stated that there exists in man a cruelty instinct. It is seldom naked, but takes cover under some form of personal or moral indignation, and one of the evils of flogging has always been that it calls forth and gives opportunity to this most anti-social state of mind in men and communities. The statement: "It gives me as much pain as it gives you" is rightly mocked at, for the terrible danger is realised of men coming to enjoy a duty that lies so close to an elemental instinct. Less clearly recognised by the lay disputants in this controversy is the connection between sexualism and flogging. In view of the history of this question that connection is clear. The Flagellants used flogging to work up in themselves emotions some of which were possibly permissible, while others were certainly most undesirable. A famous flogging master fell a victim to exactly this danger. The connection between flogging, or something similar in the name of massage, and sexual degradation is well known; and, though it is difficult to introduce these points into a general discussion they cannot be lost sight of, for they are inherent in the whole question, and though they are only associated with the more violent forms of corporal punishment, they surely cannot be dissociated from the lighter. "Spare the rod and spoil the child," usually regarded as coming from the Old Testament, is as a matter of fact from a passage by a seventeenth-century writer, in which he did indeed treat the subject as an indecent one.

A great deal of flogging goes on in the world, in many parts of it under British rule, where natives are flogged (normally to the point of drawing blood and fainting, if not worse), and the necessity for clear thinking on the whole subject is important,

**THE PUBLIC WELL-BEING**—*(continued)*.

in view of the attempts to spread it which from time to time recur. Wellington practically flogged his armies on to their victories. It was a practice of which Lord Roberts utterly disapproved, and no man can say that our soldiers and sailors to-day do not fight better than their predecessors, to whom the lash and the triangle were a regular means of discipline, or, rather, of that subservience which inferior minds regard as discipline.

